# Genome- and Transcriptome-Wide Characterization of AP2/ERF Transcription Factor Superfamily Reveals Their Relevance in *Stylosanthes scabra* Vogel Under Water Deficit Stress

**DOI:** 10.3390/plants15010158

**Published:** 2026-01-04

**Authors:** Cínthia Carla Claudino Grangeiro Nunes, Agnes Angélica Guedes de Barros, Jéssica Barboza da Silva, Wilson Dias de Oliveira, Flávia Layse Belém Medeiros, José Ribamar Costa Ferreira-Neto, Roberta Lane de Oliveira-Silva, Eliseu Binneck, Reginaldo de Carvalho, Ana Maria Benko-Iseppon

**Affiliations:** 1 Laboratório de Expressão Gênica, Agronomy Department, Federal Rural University of Pernambuco, Rua Dom Manuel de Medeiros, s/n, Recife 52171-900, PE, Brazil; cinthia.carla@ufrpe.br (C.C.C.G.N.); reginaldo.ufrpe@gmail.com (R.d.C.); 2 Laboratório de Genética e Biotecnologia Vegetal, Center of Biosciences, Genetics Department, Federal University of Pernambuco, Av. Prof. Moraes Rego, 1235, Recife 50670-901, PE, Brazil; agnes.barros88@gmail.com (A.A.G.d.B.); jessica.barboza@ufpe.br (J.B.d.S.); wilson.dias@ufpe.br (W.D.d.O.); flavia.lmedeiros@ufpe.br (F.L.B.M.); joseribamar.ferreiraneto@ufpe.br (J.R.C.F.-N.); 3 Embrapa Soja—Brazilian Agricultural Research Corporation (Embrapa), Rodovia Carlos João Strass, s/n, Acesso Orlando Amaral, Distrito de Warta, Londrina 86001-970, PR, Brazil; eliseu.binneck@embrapa.br; 4 Laboratório de Análises Genéticas, Departamento de Ciências Naturais e da Terra, Universidade do Estado de Minas Gerais-Unidade Divinópolis, 2 Andar, Bloco 1, Sala 117, Avenida Paraná, 3001, Divinópolis 35501-170, MG, Brazil; lane.roberta@gmail.com

**Keywords:** transcriptomics, abiotic stress, legume crops, RNA-seq, gene expression

## Abstract

*Stylosanthes scabra*, a legume native to the Brazilian semiarid region, exhibits remarkable drought tolerance and represents a valuable model for studying molecular adaptation in legumes. Transcription factors of the AP2/ERF superfamily play central roles in plant development and stress response. This study aimed to identify and characterize AP2/ERF genes in *Stylosanthes scabra* and to analyze their transcriptional response to root dehydration. Candidate genes were identified through a Hidden Markov Model (HMM) search using the AP2 domain profile (PF00847), followed by validation of conserved domains, physicochemical characterization, prediction of subcellular localization, phylogenetic and structural analyses, and functional annotation. A total of 295 AP2/ERF proteins were identified and designated as *Ssc*AP2/ERF, most of which were predicted to be localized in the nucleus. These proteins exhibited a wide range of molecular weights and isoelectric points, reflecting structural diversity, and were classified into four subfamilies: AP2, ERF, DREB, and RAV. Functional annotation revealed predominant roles in DNA binding and transcriptional regulation, while promoter analysis identified numerous stress-related cis-elements. A total of 32 transcripts were differentially expressed under 24 h of water deficit, and four selected genes had their expression patterns validated by qPCR. These findings provide new insights into the AP2/ERF gene subfamily in *Stylosanthes scabra* and lay the groundwork for future biotechnological approaches to enhance stress tolerance in legumes.

## 1. Introduction

*Stylosanthes scabra* Vogel belongs to the Fabaceae (Leguminosae) and to the genus *Stylosanthes*, which includes other economically important forage species, such as *S. capitata*, *S. guianensis*, and *S. macrocephala*. They are widely cultivated as pasture plants due to their high nutritional value in animal diet, adaptability to marginal soils, and importance in animal feeding, which has increased the use of *S. scabra* cultivation in recent years [1,2]. Another advantage of cultivating *S. scabra* is its ability to recover degraded areas, due to its high efficiency in nitrogen fixation (especially in low-fertility soils) [3]. Within the genus, the referred species stands out for its high tolerance to dry climates and nutrient-poor soils [4].

Under environmental adversities such as drought, *S. scabra*’s ability to adapt to stressful conditions becomes even more relevant. Drought is one of the major factors limiting global agricultural production, resulting in productivity losses of 40% in several plant crops [5,6]. Other abiotic stresses including extreme temperatures, water restriction, salinity, and heavy metals soil contamination also affect plant development. In response, plants produce signaling molecules that trigger physiological, morphological, and molecular changes through signal transduction [5,6]. Signal transduction is regulated by sophisticated molecular mechanisms that include activation of Transcription Factors (TFs), which promote the expression of genes responsible for stress adaptation [7,8].

TFs are proteins responsible for gene regulation and play a crucial role in plant development and adaptation to unfavorable conditions, as they contain specific regions that bind to cis-regulatory elements in the promoters of target genes [9], allowing or blocking gene transcription [10]. The AP2/ERF superfamily is one of the largest TF subfamilies in plants and was first reported during floral development in *Arabidopsis thaliana* [11]. Studies have shown that members of this superfamily are also involved in plant growth, hormonal regulation, and response to biotic and abiotic stresses [12]. Recently, AP2/ERF TFs have been identified and characterized in several crops, such as lettuce [13], citrus [14], peanut (*Arachis hypogaea*) [15], corn (*Zea mays*) [16], strawberry (*Fragaria* x *ananassa*) [17], and soybean (*Glycine max*) [18].

AP2/ERF TFs are classified into four main subfamilies based on amino acid similarity and conservation of protein domains: AP2 (APETALA2), ERF (Ethylene-Responsive-Element-Binding protein), DREB (Dehydration Responsive Element-Binding), and RAV (Related to ABI3/VP), in addition to a group of solo proteins (unclassified). The AP2 subfamily is characterized by the presence of two AP2 domains. In turn, the ERF subfamily contains only one domain, which is subdivided into ERF and DREB according to the conserved amino acids at positions 14 and 19. When conserved valine (V14) and glutamic acid (E19) domains are present, the protein is classified as DREB, while the presence of alanine (A14) and aspartic acid (D19) defines the ERF class. Finally, the RAV subfamily is identified by the presence of a B3 domain, in addition to the AP2 domain [19,20].

The AP2 domain encodes a DNA-binding sequence of approximately 60 amino acids, enabling protein interactions with cis-regulatory elements, such as the GCC box (AGCCGCC) and dehydration-responsive elements (DRE), which contain the central motif A/GCCGAC, located in the promoters of target genes [20]. DREB proteins bind to A/GCCGAC sequences of the ethylene-responsive element and act mainly in gene regulation against abiotic stresses, such as drought, salinity, and low temperatures. In addition, they interact with the DRE/CRT element, regulating drought-responsive genes and responding to plant hormones, including ethylene (ET) and abscisic acid (ABA) [21]. ERF proteins, on the other hand, can bind to the GCC box region, regulating the expression of pathogenesis-related genes (Pathogenesis-Related, PR) and mediating plant-pathogen interaction [22].

Although studies on AP2/ERF TFs are widely explored, research using molecular approaches and bioinformatic tools in *S. scabra* and other species of the genus *Stylosanthes* under stress conditions remains scarce [23,24,25,26]. Recently, the genome of *S. scabra* was sequenced and assembled by our research group [27], providing an essential database for molecular studies in this species. In addition, the transcriptome obtained from a 24 h water restriction assay was also sequenced [23], providing crucial information for the development of the present work.

In this study, we characterized the AP2/ERF transcription factors in *S. scabra* using genomic and transcriptomic approaches. Through transcriptomic analyses, we identified promising genes responsive to water deficit, providing valuable insights for the development of cultivars better adapted to adverse environmental conditions and enhancing our understanding of the adaptive mechanisms underlying the resilience of this important forage legume.

## 2. Results

### 2.1. Identification, Classification, and Analysis of AP2/ERF Transcription Factors in S. scabra Genome

A total of 295 primary sequences of AP2/ERF transcription factors with complete AP2 domains were identified in the *Stylosanthes scabra* genome. The loci and respective protein sequences were renamed beginning with *Ssc*AP2/ERF01 up to *Ssc*AP2/ERF295 according to their alignment in the Neighbor Joining (NJ) tree.

**Figure 1:**
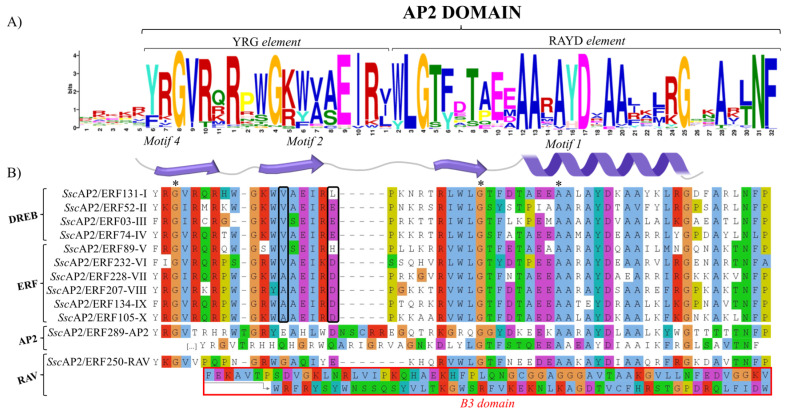
(**A**) Motifs identified within the AP2 domain of the *S. scabra* genome highlighted the conserved YRG and RAYD elements, with motifs 1, 2, and 4 collectively forming the AP2 domain across all sequences. (**B**) Conserved AP2 domain in each group of the AP2 subfamily. The black rectangles highlight the conserved amino acid positions 14 and 19. Asterisks indicate residue conservation across the 295 *Ssc*AP2/ERF proteins.

The AP2 domain of *S. scabra* was divided into two regions, known as the YRG and RAYD elements (Figure 1A), which are reported in the literature as highly conserved within this superfamily. The YRG element corresponds to a basic, well-conserved region found in most members of the AP2/ERF superfamily and defines the beginning of the domain. However, *Ssc*AP2/ERF sequences showed significant divergence among the groups. Upon analyzing the alignment of the *Ssc*AP2/ERF sequences, we observed that most sequences lacking the conserved Y (tyrosine) had an F (phenylalanine) in its place, while the G (glycine) residue was conserved in all 295 proteins (Figure 1B).

The RAYD element contained a well-conserved region responsible for forming an α-helix, which is important for the structure and function of the AP2 domain. Additionally, two residues were invariant in all the sequences: an alanine (A) and a glycine (G), the latter of which has been shown to be important for AP2 function.

The conservation of amino acids at positions 14 and 19 determines whether the protein belongs to the DREB (105 members) or ERF subfamily (144 members). In DREB, valine (V) and glutamic acid (E) are conserved, while in ERF, alanine (A) and aspartic acid (D) are conserved, respectively. Examination of the sequence alignment revealed that these residues were conserved in most of the analyzed proteins. In the DREB subfamily, valine was present in all 105 members, while glutamic acid was conserved in only 63 members. In the remaining sequences, amino acid substitutions were observed, with leucine (L) being the most frequent replacement (Supplementary Material, Figure S1).

In the ERF subfamily, the conservation of alanine (A) and aspartic acid (D) was expected. Except for five members that showed a substitution of aspartic acid with histidine (D/H), all others maintained conservation of this residue. Additionally, 32 members exhibited substitutions at position 14 with various amino acids, with valine (V) being the most common (Supplementary Material, Figure S2). The RAV subfamily contains only one AP2 domain. In *S. scabra*, the YRG motif was replaced by either YKG or FKG. The WLG motif was conserved across all sequences, whereas in the RAYD motif, the arginine (Arg) was substituted by either lysine (K) or methionine (M).

**Figure 2:**
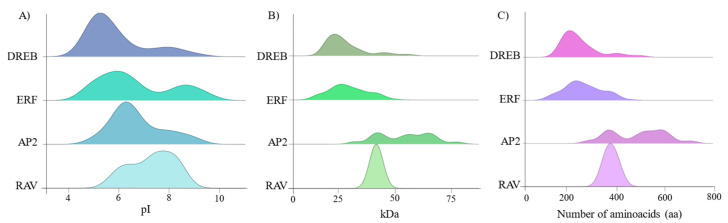
Density plot of the isoelectric point values (**A**), molecular weight (**B**), and number of amino acids (**C**) of the 295 *Ssc*AP2/ERF proteins.

In silico physicochemical analyses of the *Ssc*AP2/ERF proteins revealed their diversity in various parameters. The observed isoelectric point ranged from 4.2 to 9.5, with most proteins showing acidic affinity (73%), in addition to 18% with neutral pH and 9% with alkaline pH (Figure 2A). Their number of amino acids and molecular weight ranged from 103 to 711 and from 11.83 to 78.61 kDa, respectively (Figure 2B,C).

Most *Ssc*AP2/ERF proteins (282) were predicted in the nucleus. The remaining were found in the chloroplast (12) and mitochondrion (1). The GRAVY value determined by the hydropathy index of the amino acids indicated that the *Ssc*AP2/ERF proteins have a hydrophilic nature (GRAVY < 0) (Supplementary Material, Table S1).

### 2.2. Neighbor Joining (NJ) Analysis, Gene Structure, and Identification of Conserved Motifs

**Figure 3:**
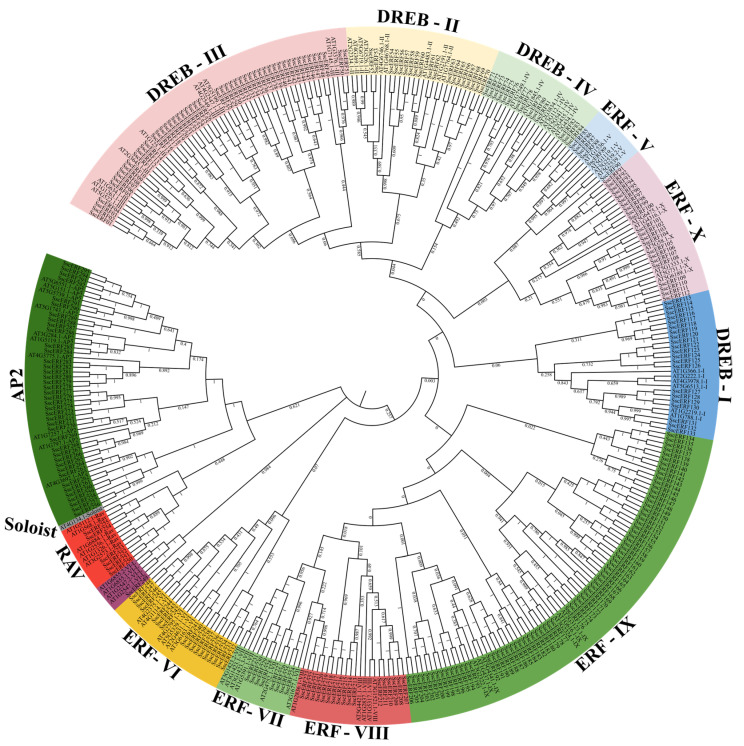
Neighbor-Joining (NJ) tree of AP2/ERF proteins from *Stylosanthes scabra* (1000 replicates). Different colors represent subfamilies and groups. Number at the base of clades indicate bootstrap values.

The NJ tree generated using AP2/ERF sequences from *S. scabra* and *A. thaliana* showed the gathering of four major subfamilies: AP2, ERF, DREB, and RAV. The AP2 subfamily comprises 40 proteins containing two conserved AP2 domains. Six proteins containing both the AP2 and B3 domains were classified in the RAV group. Finally, 144 proteins were grouped into the ERF subfamily and 105 into the DREB subfamily (Figure 3).

The ERF proteins were subdivided into groups VI, VI-L, VII, VIII, IX, X, and Xb-L according to the presence of specific conserved motifs, while DREB proteins were subdivided into groups I, II, III, and IV (Figure 3). The number of members in each group ranged from 6 to 73 in groups V and IX, respectively (Figure 3); however, a tendency towards a larger number of members was observed in group IX when compared to other plant species (Figure 3).

**Figure 4:**
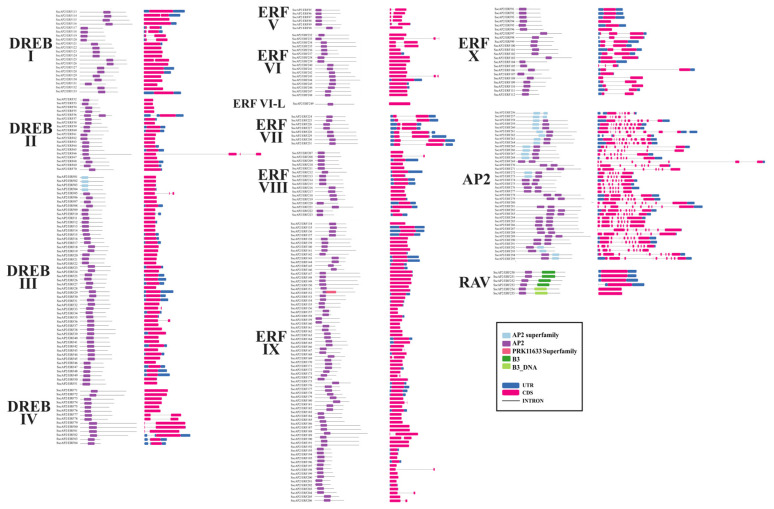
Gene structures and domains of AP2/ERF proteins from *S. scabra*. Columns on the left indicate the conserved domains, while columns on the right represent gene structures. Different colors represent domains and gene structures. In the right column, the UTR regions are shown in blue, exons in pink, and introns as black lines.

A detailed analysis of gene structure was also performed for each group. Our investigation revealed that 95 genes contained at least one intron (Figure 4) and that gene structures were highly similar within each group. Among the 105 genes belonging to the DREB subfamily (groups I, II, III, and IV), 14 contained between one and three introns. Most of these genes were present in groups DREB-I, DREB-III, and DREB-IV, with only one sequence containing a single intron present in group DREB-II (Figure 4).

In the ERF subfamily (V–X), out of the 144 identified genes, 40 had between 1 and 2 introns, with groups ERF-IX and ERF-X being the most representative, containing nine and fourteen members, respectively (Figure 4). However, all members of group ERF-VII contained one intron. Among the 40 members of the AP2 subfamily, all included at least four introns and up to nine. On the other hand, none of the six genes belonging to the RAV subfamily contained introns (Figure 4).

The analysis of conserved motifs showed that motifs 1, 2, and 4 are present in all AP2/ERF proteins of *S. scabra*, suggesting that they are essential components of the AP2 domain structure (Figure 1A). Motifs 18, 20, 24, and 22 were specific to groups DREB-IV, ERF-V, ERF-VII, and ERF-IX, respectively. On the other hand, motifs 3, 5, 13, and 19 were identified exclusively in the AP2 subfamily, as were motifs 16 and 17, which were found only in proteins of the RAV group. Overall, it was observed that motifs tend to be conserved within groups or according to phylogenetic proximity. Interestingly, there was considerable variation among the four groups of the DREB subfamily, with motif 18 being identified as exclusive to group DREB-IV (Supplementary Material, Figure S3).

The ERF subfamily had a greater abundance of motifs when compared to the others, as it had four exclusive motifs identified in groups ERF-V, ERF-VII, ERF-VIII, and ERF-IX (motifs 20, 22, 24, and 25, respectively). The EDLL motif, typically associated with transcriptional activation in ERF proteins, was identified in groups ERF-VIII and ERF-IX. In contrast, the EAR repression motif (DLNXP), known for its involvement in transcriptional suppression, was observed in groups ERF-VIII and ERF-X, suggesting the coexistence of both activation and repression regulatory elements within group ERF-VIII members (Supplementary Material, Figure S4).

The AP2 and RAV subfamilies also exhibited conservation of distinct motifs, with motifs 3, 5, 13, and 19 found exclusively in the AP2 subfamily. The presence of motif 3 suggests that it corresponds to a region within the second AP2 domain, as it contains the RAYD element in its sequence—a hallmark feature of members of this subfamily (Supplementary Material, Figure S5). The RAV subfamily exhibited six different motifs, two of which were exclusive to this group and related to its B3 domain.

### 2.3. Mechanisms of SscAP2/ERF Genomic Expansion

**Figure 5:**
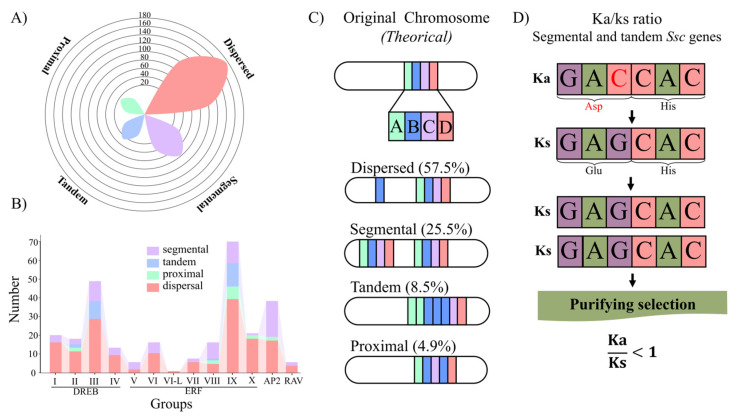
Gene duplication mechanisms and Ka/Ks ratio of *Ssc*AP2/ERF genes from *S. scabra*. (**A**) Number of genes according to each type of gene duplication. (**B**) Types of duplications occurring in each subgroup. (**C**) Ka/Ks ratio for genes duplicated by segmental and tandem duplication. (**D**) Schematic illustration of each type of gene duplication and the percentage found among *Ssc*AP2/ERF genes, the nucleotide “C” highlighted in red marks the position that will undergo mutation in the sequence below.

All 295 *Ssc*AP2/ERF genes underwent some duplication events throughout evolution; however, dispersed and segmental duplications were predominant (Figure 5A). Groups DREB-III, ERF-IX, and AP2 were the most abundant, with most of their duplications arising from dispersed and segmental events (Figure 5B). In turn, DREB-I, DREB-IV, ERF-V, ERF-VI, ERF-VII, and RAV exhibited only two types of duplication: dispersed and segmental. Regarding duplication events, dispersed duplication accounted for 57.5%, segmental for 25.5%, tandem for 8.5%, and proximal duplication for 4.9% (Figure 5C).

The selective pressure analysis showed that the Ka/Ks ratios of genes duplicated by segmental and tandem events were mostly subjected to purifying selection, with all AP2/ERF gene pairs presenting Ka/Ks < 1. Among them, several pairs exhibited strong purifying selection (Ka/Ks < 0.2), including *Ssc*AP2/ERF01–*Ssc*AP2/ERF25, *Ssc*AP2/ERF28–*Ssc*AP2/ERF07, *Ssc*AP2/ERF163–*Ssc*AP2/ERF162, *Ssc*AP2/ERF58–*Ssc*AP2/ERF56 and *Ssc*AP2/ERF199–*Ssc*AP2/ERF166 (Figure 5D).

### 2.4. SscAP2/ERF Gene Ontology

**Figure 6:**
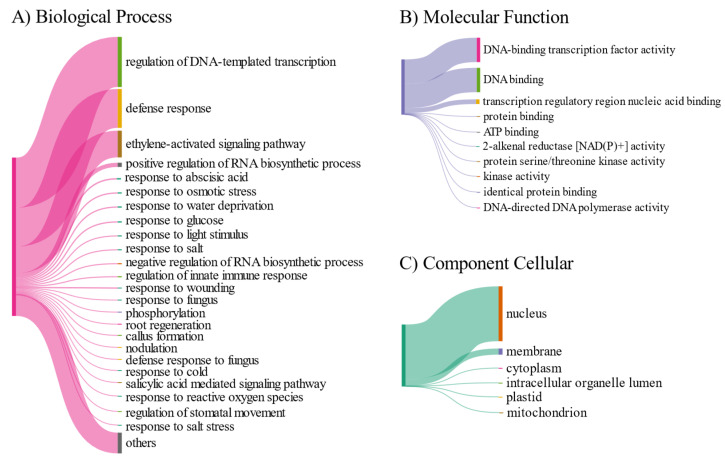
Functional annotation of AP2/ERF proteins from *S. scabra* based on Gene Ontology (GO) terms. (**A**) Biological processes. (**B**) Molecular function. (**C**) Cellular component.

The GO term analysis indicated that AP2/ERF proteins from *S. scabra* were involved in significant biological processes. All *Ssc*AP2/ERF were associated with the regulation of DNA-templated transcription (GO:0006355), as was expected for transcription factors. Defense response (GO:0006952) was the second most representative GO term, followed by ethylene-activated signaling pathway (GO:0009873) (Figure 6A).

Regarding molecular function, the most representative terms were DNA-binding transcription factor activity (GO:0003700) and DNA binding (GO:0003677), which are involved in regulating gene transcription modulation and DNA interaction (Figure 6B). Additionally, in the cellular component category, most proteins were annotated in the nucleus (GO:0005634), followed by the plasma membrane (GO:0016020), suggesting that these proteins may act as receptors in recognizing stimuli at the membrane (Figure 6C).

### 2.5. Analysis of Cis-Regulatory Elements of AP2/ERF Genes

**Figure 7:**
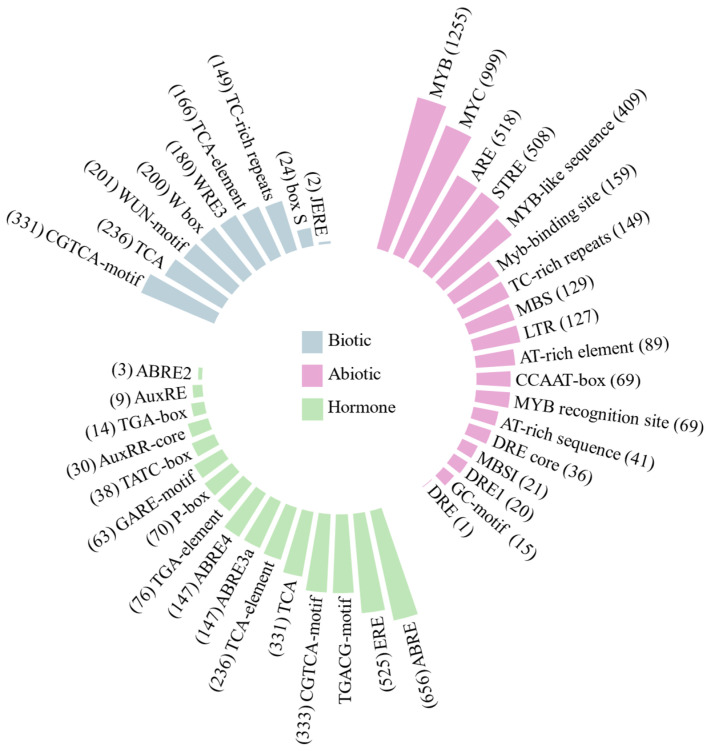
Circular bar plot represents the distribution of *cis*-regulatory elements related to abiotic stress (pink), hormone signaling (green), and biotic stress (blue). Bar height indicates the frequency of cis-regulatory elements within the categories.

A total of 118 cis-regulatory elements were identified in the promoter regions of the AP2/ERF genes from *S. scabra*, which were categorized according to their functions. Most were annotated as elements responsive to light, development, abiotic and biotic stresses, hormones, as well as elements related to promoters. Regarding cis-elements related to biotic stresses, the CGTCA-motif was identified, which is involved in the response to methyl jasmonate (MeJA), a plant hormone associated with environmental stress responses and regulation of defense-related genes and secondary metabolite production. Additionally, TCA, W-box, and Wun elements responsive to wounding and pathogens were found (Figure 7).

Among the cis-elements responsive to abiotic stresses (Figure 7), MYB and MYC motifs were the most frequently identified. These elements are known to participate in water deficit response, along with ARE and STRE motifs, which are associated with adaptation to adverse environmental conditions, including drought and salt stress. Additionally, the cis-elements DRE, DRE1, and DRE-core—also relevant to drought response—were identified, although at lower frequencies in this analysis. Regarding hormone-responsive cis-elements (Figure 7), ABRE (ABA-Responsive Element) and ERE (Ethylene-Responsive Element) were the most representative, both crucial in plant responses to osmotic stress. The TGACG and CGTCA elements were also frequent and responsive to MeJA.

The analysis of transcription factor binding sites (TFBS) revealed three transcription factors (C2H2 zinc finger, MYB, and Basic helix-loop-helix-bHLH) with significant e-value and *p*-value scores (Supplementary Material, Table S2).

### 2.6. Structural Models of SscAP2/ERF Proteins

**Figure 8:**
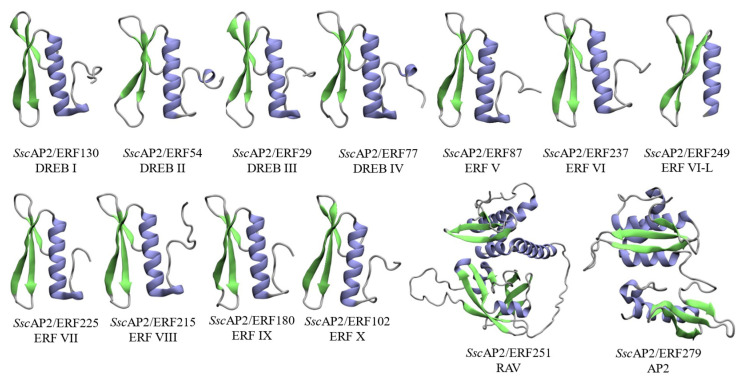
Predicted three-dimensional structures of representative proteins from the AP2/ERF superfamily in *S. scabra*. Examples are shown for the DREB-I, DREB-II, DREB-III, DREB-IV, ERF-V, ERF-VI, ERF-VII, ERF-VIII, ERF-IX, ERF-X, RAV, and AP2 subfamilies. α-helices are shown in purple, β-sheets in green, and loop regions in grey.

The predicted three-dimensional structures of representative members of the AP2/ERF superfamily in *S. scabra* revealed the characteristic AP2 domain composed of β-sheets (in green) and α-helices (in lilac) (Figure 8), with conformational variations among the subfamilies. The DREB-I, DREB-II, DREB-III, DREB-IV, and ERF-V, ERF-VI, ERF-VII, ERF-VIII, ERF-IX, and ERF-X groups displayed structural similarities but with subtle differences in loop regions and helix orientations, suggesting potential functional diversification in DNA-binding specificity. In contrast, members of the RAV and AP2 subfamilies exhibited more complex structural arrangements. Members of the RAV combine an AP2 domain and a B3 domain, reflecting their functional versatility and regulatory role in broader processes. In turn, the *Ssc*AP2/ERF279, representative of AP2 subfamily members, presents a double AP2 domain, indicating greater structural complexity and potential to interact with different DNA sequences (Figure 8).

Theoretical models from *Ssc*AP2/ERF proteins presented validation metrics compatible with quality parameters widely accepted for three-dimensional protein models [28]. The analysis using ProSA showed Z-score values ranging from −8.07 (ID 251) to −2.98 (ID 249). PROCHECK results revealed that all models had more than 92% of residues located in favorable regions of the Ramachandran plot, with particular emphasis on models ID 87 and 237, which reached 98.0% of residues in energetically favorable conformations. High PROCHECK values (>96%) were observed for models with IDs 54, 87, 130, 180, 215, and 237. Regarding QMEAN, values ranged from 0.70 (ID 215) to 0.80 (ID 180) (Supplementary Material, Table S4). The complementary analysis using QMEANDisCo confirmed the structural consistency and robustness of the obtained models.

### 2.7. Differential Expression of SscAP2/ERF Genes Under Water Deficit Stress

**Figure 9:**
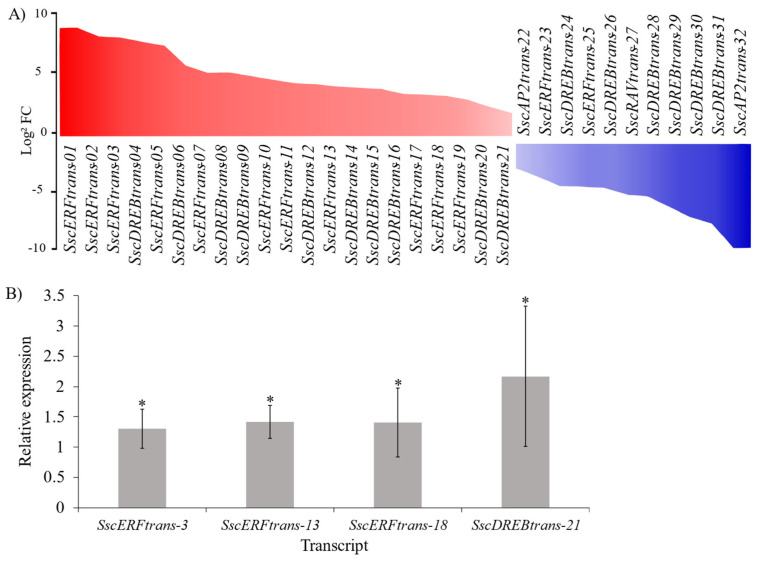
Differential expression of *Ssc*AP2/ERF transcripts under water deficit stress. (**A**) Heatmap-like bar plot showing the Log2 fold-change (Log_2_FC) of 32 differentially expressed transcripts after 24 h of water deficit. Red bars indicate upregulated transcripts and blue bars indicate downregulated transcripts. (**B**) Relative expression analysis of selected transcripts (*Ssc*AP2/ERFtrans-3, *Ssc*AP2/ERFtrans-13, *Ssc*AP2/ERFtrans-18, and *Ssc*DREBtrans-21) by qPCR, confirming the transcriptome expression patterns. Bars represent mean relative expression ± standard error (SE). Asterisks (*) above bars indicate statistically significant differences at *p* < 0.05.

A total of 32 differentially expressed *Ssc*AP2/ERF transcripts were identified (Figure 9A) in the *S. scabra* transcriptome under 24 h of water deficit, with 21 being upregulated and 11 downregulated, showing modulation values (Log2FC) ranging from 9.3 to –9.0. *Ssc*ERFtrans-01 and *Ssc*ERFtrans-02 stood out among the others, as they exhibited strong upregulation with Log_2_FC values above 8.3. On the other hand, *Ssc*AP2trans-32 and *Ssc*DREBtrans-31 were strongly downregulated, with Log_2_FC values below –6.0, indicating a possible suppression of functions associated with stress tolerance. It is also noteworthy that none of the RAV family members were upregulated under water deficit condition; instead, only a single RAV gene exhibited downregulated expression.

Relative expression analysis by qPCR confirmed the transcriptomic patterns for *Ssc*ERFtrans-3, *Ssc*ERFtrans-13, *Ssc*ERFtrans-18, and *Ssc*DREBtrans-21 (Figure 9B), with all evaluated genes showing increased expression under stress conditions compared to the control.

## 3. Discussion

The AP2/ERF transcription factor (TF) superfamily is one of the largest in plants and plays a crucial role in regulating various processes, including plant growth and development, as well as responses to abiotic stresses (drought, salinity, heat, and flooding) and biotic stresses (infection by bacteria, fungi, and viruses) [29,30,31]. In recent years, the number of available genomes in databases has significantly increased and supported many studies on the functional and evolutionary relationships of AP2/ERF TFs. Examples of this scenario are studies developed in *A. thaliana* (147 genes) and rice (*Oryza sativa*, 139 genes) [19], pearl millet (*Pennisetum glaucum*, 167 genes) [32], sweet cherry (*Prunus avium* L., 50 genes) [33], wild strawberry (*Fragaria vesca* L., 86 genes) [34], and wheat (*Triticum aestivum* L., 517 genes) [29]. In this study, we identified 295 AP2/ERF proteins in *S. scabra*, which corresponds to 0.24% of the species’ entire conceptual proteome. The expansion in the number of members in the *Ssc*AP2/ERF superfamily is possibly associated with its allotetraploid genome [35].

The conserved domain analysis of *Ssc*AP2/ERF proteins allowed classification into four subfamilies: AP2 (containing two AP2 domains), ERF and DREB (with a single AP2 domain), and RAV (with a single AP2 domain plus an additional B3 domain) [12]. The primary distinction between the DREB and ERF subfamilies lies in the conserved amino acids at positions 14 and 19 within the AP2 domain. ERF proteins typically exhibit A14 and D19, while DREB proteins contain V14 and E19 [19]. The presence of V14 and E19 in the binding domain plays an important role in recognizing and interacting with the DRE regulatory element [20]. In our study, valine (V) conservation at position 14 was verified for DREB members. However, frequent substitution of glutamic acid (E19) by leucine (L) was observed in several sequences, suggesting that position 14 has greater functional importance for DREB proteins. The predominance of valine conservation and the E/L substitution have been reported in other studies [36,37]. Recent studies suggest that the methyl group at position 14 is essential for protein interaction with the cis DRE, while the carboxyl group at position 19 is not fundamental for this binding. Substitution of V14 by G14 eliminates interaction with DRE, highlighting the critical role of V14 in recognition. In contrast, substitution of E19 by Q19 maintains binding capacity, suggesting that the carboxyl group at position 19 may not play a key role in this process [38].

Our results showed two important conserved elements—YRG and RAYD—in the *Ssc*AP2 domain. The YRG element, composed of three β-sheets, primarily functions in DNA binding at cis-regulatory regions, and it is essential for target sequence recognition. The RAYD element forms an α-helix that is responsible for specific DNA interactions and mediates interactions with other proteins, contributing to the efficient regulation of molecular processes [39]. Overall, high conservation of these elements was observed in *S. scabra* AP2/ERF proteins, with glycine (G) in the YRG element present in all proteins. However, tyrosine (Y) and arginine (R) were mostly substituted by FK or FR, a pattern also reported for other plant species such as *Zanthoxylum bungeanum* [40] and *Taxus chinensis* [41].

Gene structure analysis showed that proteins within the same group share similar exon-intron patterns. For example, AP2 proteins had a high number of introns (4 to 9). In contrast, ERF, DREB, and RAV subfamily members had few or no introns, which may be related to evolutionary adaptations that enhance gene expression efficiency [42]. All six members of the RAV subfamily lacked introns, a structural pattern characteristic of this subfamily, also observed in other plant species such as *Eschscholzia californica* and *Zea mays* [16,43].

It is well-established that intron-poor genes are derived from originally intron-rich genes and have undergone evolutionary adaptation, resulting in greater efficiency in stress responses. This feature is associated with easier transcription and the absence of alternative splicing, allowing more rapid expression [44]. Studies in *A. thaliana* and rice revealed that the percentage of differentially expressed genes (DEGs) from the AP2 subfamily without introns was significantly higher compared to intron-rich DEGs (*p* < 0.01), suggesting that intron-less genes play more relevant roles in abiotic stress responses such as drought and salinity [44]. However, this pattern could not be assessed in the present study, as no AP2 genes were up-regulated under the evaluated conditions, with AP2 members being exclusively down-regulated. The intron distribution in *Ssc*AP2/ERF suggests that ERF, DREB, and RAV members are more conserved across species and that structural variation among these subfamilies indicates possible evolutionary differentiation, which led to differences in gene sequences.

Conserved domains and motifs in transcription factors play an essential role in gene function and transcriptional regulation [45,46]. Some motifs identified in *Ssc*AP2/ERF proteins have functions that are well described in the scientific literature. For example, the EAR motif (consensus sequence LxLxL or DLNxxP) corresponds to a transcriptional repression motif located in the C-terminal region, widely found in plants [47]. In our study, the EAR motif was identified in 21 members, predominantly in group ERF-VIII, highlighting its representativeness in the AP2/ERF subfamily [32,33]. Genes containing the EAR motif may act as transcriptional repressors of ABA-responsive genes. This impacts essential physiological processes under plant water stress [48]. Our study also identified the EDLL motif, known to act as a transcriptional activator capable of partially overcoming repression induced by the EAR motif [49]. For instance, the EDLL motif of *Musa acuminata* potentially activates the expression of ethylene-mediated genes, promoting starch degradation, playing an important role in fruit ripening [50].

Neighbor-Joining analysis classified AP2/ERF proteins into 11 clades, with groups DREB-III and ERF-IX encompassing most proteins. Guo et al. [51] suggested that the AP2/ERF groups DREB-III and ERF-IX were convergently expanded in eudicots through whole-genome duplications (WGD) and tandem duplications (TD), particularly during periods of global cooling. Group ERF-IX included most *Ssc*AP2/ERF proteins. Previous studies have shown that members of this group confer resistance to the fungus *Stemphylium lycopersici* in interaction with other genes [52], resistance to *Botrytis cinerea* [53], and positively regulate ethylene to confer resistance against the necrotrophic bacterium *Pectobacterium carotovorum* [54]. Group ERF-VII included only eight members; however, all presented the N-terminal motif MCGGAI(I/L) [19], which is related to drought tolerance in *Ginkgo biloba* [55] and hypoxia response in *Arabidopsis*, rice, and *Brachypodium* [56].

Regarding the expansion mechanisms of these genes, *Ssc*AP2/ERF genes expanded mainly through dispersed and segmental duplications. A study comparing plants and algae also found that both types of duplications contributed most to the expansion of the AP2/ERF superfamily, indicating these duplications were fundamental for acquiring new adaptive functions within the subfamily [57].

Tandem and proximal duplications, as some here identified, represent biologically crucial paths for adaptive evolution of the AP2/ERF superfamily in *S. scabra*. The physical proximity of duplicated copies—adjacent in tandem duplications mainly caused by unequal crossing-over [58], or closely separated in proximal duplications [59]—facilitates divergence. This gene organization in the genome facilitates gene neofunctionalization or division of functions among copies, helping the gene subfamily to adapt and perform more specialized roles.

Purifying selection (Ka/Ks < 1) in duplicated AP2/ERF genes in *S. scabra* was predominant, suggesting the preservation of essential functions. This may be due to the fact that gene duplication, together with this conservative selective pressure, has been crucial for the functional evolution and stability of this gene subfamily, as such selective pressure acts to maintain the biological activity of the genes [60].

Cis-regulatory elements are short motifs present in DNA sequences located in gene promoter regions. They are recognized by transcription factors and play a fundamental role in controlling transcription and regulating gene expression [61]. We identified many cis-regulatory elements in *S. scabra* genes, mostly related to light and development, which are essential processes for plants. MYB, MYC, ARE, and STRE elements were most abundant in the abiotic stress category. Notably, the presence of DRE and ABRE elements suggests a response to abiotic stress through ABA-mediated hormonal pathways [62]. The *ZmEREB160* gene was characterized as an important regulator of drought resistance molecular mechanisms as it contained DRE and ABRE cis-elements in its promoter region [63]. Similarly, three *S. scabra* genes have these elements, suggesting their role in tolerance to water deficit stress.

Our results indicate that AP2/ERF genes are regulated by diverse transcription factors, playing multifunctional roles in processes such as responses to abiotic and biotic stresses, development, and hormonal regulation. Zhu et al. [64,65] demonstrated a close relationship between AP2/ERF and MYB genes in *Vitis amurensis* ‘Shuang You’, consistent with the associations observed in the present study. The *VaMYB306* gene, interacting with *VaERF16*, conferred greater resistance of grapevine to the fungus *Botrytis cinerea*. Additionally, Lv et al. [66] found that the interaction between *VaMYB4a* and *VaERF054-like* increased species tolerance to cold.

Protein modeling of *S. scabra* AP2/ERF members showed high precision in predicting their 3D-structures. The models generated were consistent with experimentally validated models, as evidenced by their similarity with structures available in the Protein Data Bank. Protein structural complexity increased as the number of domains expanded, also increasing the number of motifs, indicating that the structure is directly related to the number of motifs present. Although the ERF group showed high similarity, we believe that the observed structural variations may reflect the diversity of their biological functions [67]. The Z-score values obtained with ProSA fell within the expected range for experimentally determined proteins, indicating good global quality of protein folding. Likewise, the high proportion of residues in favorable regions of the Ramachandran plot (>92%), with some models reaching up to 98%, reinforces the stereochemical suitability of the generated structures. QMEAN values (0.70–0.80) also indicate good agreement with high-quality reference structures, while QMEANDisCo analysis confirmed the structural consistency and robustness of the predictions, supporting the reliability of the generated models [68].

GO-enrichment suggests a central role of *Ssc*AP2/ERF proteins in stress response. The presence of the “defense response” (GO:0006952) GO term supports evidence that ERF proteins can actively participate in pathogen response. In addition, the enrichment of “response to water deprivation” (GO:0009414) highlights the involvement of *Ssc*AP2/ERF genes in abiotic stress adaptation, particularly under drought conditions. This observation is consistent with recent findings that AP2/ERF transcription factors orchestrate regulatory networks that enhance plant tolerance to abiotic stresses by activating protective stress-responsive genes [65]. Furthermore, the ethylene signaling pathway (GO:009873) aligns with the literature, which demonstrates that ERF factors serve as mediators of the ethylene response, modulating target gene transcription and regulating ethylene synthesis in plant tissues [69]. Annotation of some genes to the cell membrane (GO:0016020) suggests a role in environmental signal perception, besides predominant nuclear localization (GO:005634), typical of transcription factors.

AP2/ERF transcription factors have been widely reported to be involved in drought and salinity stresses [70,71,72]. In our study, 32 AP2/ERF transcripts were differentially expressed in root RNA-Seq libraries of *S. scabra* under 24 h of water deficit. Out of these, 21 were up-regulated, where ten of them belonged to the DREB subfamily known for its essential role in regulating drought tolerance. DREB factors directly interact with specific cis-regulatory elements, such as the Dehydration-Responsive Element (DRE) located in the promoters of target genes and responsible for inducing expression of genes related to water stress adaptation [73]. Under drought stress conditions, roots are the most affected tissues. On the other hand, they serve as primary sensors of water deficit, initiating regulation of gene expression in response to water scarcity. Thus, roots play a crucial role in activating molecular signaling mechanisms that control metabolic pathways involved in drought-adaptive responses, enabling plants to adjust metabolism and maintain homeostasis under adverse conditions [74].

This proportion of 32 DEGs is consistent with previous transcriptomic studies, in which only a subset of AP2/ERF members exhibit transcriptional modulation at specific time points [75] AP2/ERFs are known to display strong tissue- and time-specific expression and diversified functions, so many family members respond in other organs (e.g., leaves) [76], or at earlier/later drought stages than the 24 h root sampling used here. In *Adiantum nelumboides*, Wu et al. [77] identified 163 AP2/ERF genes and, based on transcriptome data under drought stress, found that 22 of them were differentially expressed. This proportion of responsive genes is consistent with findings in other species, where only a minority of AP2/ERF members respond transcriptionally to a single stress condition [77].

Transgenic and gene overexpression studies have shown the involvement of AP2/ERF genes in enhancing tolerance to biotic and abiotic stresses. For example, overexpression of *OsERF71* and *OsERF48* in transgenic rice plants resulted in drought-tolerant phenotypes [55,78]. The *GmDREB1* gene has been reported as conferring drought tolerance in soybean [79] and wheat [31]. RAV genes, in turn, show more variable roles. Some studies report positive effects on drought tolerance [80], while others show negative functions, such as the silencing of NtRAV4, which increased water retention and antioxidant activity in tobacco [81]. However, in this study, the absence of induced RAV genes and the repression of a single member is particularly noteworthy, as this unique downregulated gene may reflect a suppressive role during dehydration, reinforcing its distinct regulatory behavior compared with the induced DREB genes.

Thus, the *Ssc*AP2/ERF genes identified in *S. scabra* show great potential for biotechnological applications and, therefore, can be explored in breeding programs involving transgenics or gene editing for developing crops more resistant to water stress.

## 4. Materials and Methods

### 4.1. Plant Material and Experiments

Briefly, the experiment was conducted at the Embrapa station, located in Petrolina (Pernambuco). *S. scabra* plant material was collected in the Caatinga region and belongs to the Active Germplasm Bank of the State University of Bahia (UNEB—12°57′7″ S 38°27′1″ W) [23]. The Caatinga vegetation is characterized by a seasonally dry tropical ecosystem dominated by thorny shrubs, small deciduous trees, cacti, and other drought-adapted plants. In turn, *S. scabra* is an erect allotetraploid forage legume (2n = 40) naturally adapted to seasonal drought, supporting its use as a model species for water-deficit studies. Accession 85/UNEB was propagated from 10 cm stem cuttings taken from semi-young to semi-lignified tissues at apical, median, and basal nodal positions, as routinely applied for *Stylosanthes* propagation, and transplanted into plastic bags containing an ultisol–vermiculite substrate (3:1). The cuttings were maintained in a climate-controlled greenhouse (50% shade) and irrigated twice daily. After that, the plants were transplanted into plastic pots, where they were kept under controlled conditions (temperature 30 ± 2 °C, relative humidity 60 ± 5%, and a 12 h natural-light photoperiod with a PPFD of 1.5 × 10^3^ μmol m^−2^ s^−1^) and watered daily (at 9:00 a.m.) once a day for 6 months.

Plants were grown for six months before being subjected to water deficit. Healthy plants with uniform growth were divided into two treatments: control and 24 h of water deficit, each comprising three biological replicates. Root tissues from both control and water suppression treatments were collected, immediately frozen in liquid nitrogen, and stored at −80 °C until RNA extraction, as previously described [23].

### 4.2. Total RNA Extraction, RNA-Seq Library Construction, and Differential Expression Analysis

Total RNA was isolated using the “SV Total RNA Isolation System” kit (Promega, Madison, WI, USA) according to the manufacturer’s protocol with the addition of a DNase treatment. Total RNA concentration, purity, and integrity were evaluated using the Qubit fluorometer (Thermo Fisher Scientific, Waltham, MA, USA), Nanodrop spectrophotometer (Thermo Fisher Scientific), and 1.5% agarose gel electrophoresis (80 V, 120 A for 40 min), stained with Blue-green Loading Dye (LGC Biotechnology, São Paulo, BR), respectively. After assessment with the Agilent 2100 Bioanalyzer (Agilent Technologies, Santa Clara, CA, USA), only samples with RNA Integrity Number (RIN) ≥ 8.0 were sequenced. The cDNA synthesis was performed using 1 μg of total RNA and Oligo (dT) primers, following the recommendations supplied by GoScript™ Reverse Transcription System Kit (Promega, Madison, WI, USA).

Six RNA-Seq libraries containing the biological replicates of the control and 24 h water suspension treatments were sequenced and used for transcriptome assembly [23]. *De novo* assembly of *S. scabra* RNA-Seq libraries was performed using Trimmomatic version 0.39 [82] to remove adapters and low-quality sequences. Only reads with a Phred score ≥ 30 were kept. Quality control was performed using FastQC version 0.39 (https://github.com/s-andrews/FastQC (accessed on 10 January 2023)). Transcriptome assembly was conducted using Trinity 2.0.4 [82], and candidate ORFs were identified using TransDecoder version 2.0.1 (https://github.com/TransDecoder/TransDecoder/wiki (accessed on 19 January 2023)) with only the longest ORF per transcript.

We used Trinotate version 2.0.2 (https://github.com/Trinotate/Trinotate.github.io/wiki (accessed on 23 January 2023)) for functional annotation of ORFs. This method employs multiple approaches, such as homology searches in known databases, identification of protein domains, and prediction of signal peptides. Assembly completeness was evaluated with gVolante version 2.0 [83] and the BUSCO v.5 ortholog search pipeline [84], which assesses the coverage of reference genes and sequence quality. Transcript abundance in each sample was estimated with the RSEM tool (RNA-Seq by Expectation-Maximization tool) [85], and differential expression analysis was performed using EdgeR software version 1.0 [86], where transcripts were considered differentially expressed when they presented Log2FC values < −1 and > 1, *p*-value < 0.05, and FDR < 0.05.

### 4.3. Identification, Analysis, and Classification of AP2/ERF Supersubfamily Genes in S. scabra Genome and Transcriptomes

The Hidden Markov Model (HMM) profile for the AP2 domain (Pfam ID: PF00847) was retrieved from the Pfam database (http://pfam.xfam.org/ (accessed on 12 January 2024)) and used to search the conceptual proteome of *S. scabra* (obtained for its genome [28]) as well as its translated RNA-Seq transcriptomes [24] using HMMER version 3.4, applying an e-value cutoff of < 0.05 [87]. Batch CD-Search tool (https://www.ncbi.nlm.nih.gov/Structure/bwrpsb/bwrpsb.cgi (accessed on 12 January 2024)) was used to verify the AP2 domain and, therefore, to confirm annotation of the retrieved sequences. Sequences with an intact AP2 domain were selected [88].

The corresponding primary protein sequences and respective loci were selected and renamed from *Ssc*AP2/ERF01 to *Ssc*AP2/ERF295, based on their distribution in NJ analysis and following the classification proposed by NAKANO et al. [19]. The following physicochemical parameters of *Ssc*AP2/ERF proteins were predicted: isoelectric point (http://isoelectric.org/ (accessed on 28 May 2024)), subcellular localization (http://cello.life.nctu.edu.tw/ (accessed on 28 May 2024)), number of residues and molecular weight (https://www.bioinformatics.org/sms2/protein_mw.html (accessed on 28 May 2024)), and GRAVY (Grand Average of Hydropathicity) index (https://www.bioinformatics.org/sms2/protein_gravy.html (accessed on 28 May 2024)).

### 4.4. Multiple Sequence Alignment and Phenetic Analysis

Multiple alignment of full-length AP2/ERF sequences was performed using ClustalW algorithm (based on ClustalW version 2.0) in MEGA X with default parameters [89]. AP2/ERF sequences from *A. thaliana*, available in the TAIR database (https://www.arabidopsis.org/ (accessed on 12 March 2024)), were also included in the comparisons. Neighbor-Joining analysis was carried out using MEGA X [89] with a bootstrap test of 1000 replicates. The resulting dendrogram was visualized and edited using Interactive Tree of Life (iTOL) (https://itol.embl.de/ (accessed on 18 March 2024)).

### 4.5. Gene Structure and Conserved Motifs

The analysis of conserved motif distribution was performed using the Multiple EM for Motif Elicitation (MEME) Suite v. 5.5.5 (https://meme-suite.org/meme/tools/meme (accessed on 5 June 2024)), setting the maximum number of motifs to 25, using the Zero or One Occurrence Per Sequence (zoops) distribution mode, with a minimum motif width of 6 and a maximum of 50. The gene structures of the *Ssc*AP2/ERF proteins were generated using the Gene Structure View tool in TBtools version 2.224 [90].

### 4.6. Gene Duplication

The gene duplication analysis of *Ssc*AP2/ERF genes in the *S. scabra* genome was performed using MCScanX tool version 1.1.11 [91]. Syntenic regions were detected by aligning the complete proteome to itself (BLASTP 2.2.28+), with an e-value cutoff of 1 × 10^−5^. This action generated a similarity file, along with the gene coordinates were used to identify and classify gene duplication events in the *S. scabra* genome. The *Ssc*AP2/ERF genes were extracted from the output file and classified according to their duplication type using the duplicate_gene_classifier script provided by the tool. For Ka/Ks estimation of segmental duplicates, the “add_ka_and_ks_to_collinearity.pl” script was employed. For tandem duplicates, CDS were translated using Transeq (EMBOSS), aligned with MAFFT, and converted into codon-aware format using PAL2NAL. Ka and Ks values were calculated with KaKs_Calculator v2.0.1 (https://github.com/kullrich/kakscalculator2 (accessed on 15 February 2025)), applying the Li (YN) model. The resulting Ka, Ks, and Ka/Ks ratios were compiled for downstream analyses.

### 4.7. Gene Ontology, Cis-Regulatory Element Analysis, and DNA-Binding Site Prediction

Functional annotation and Gene Ontology (GO) categories associated with the *Ssc*AP2/ERF proteins was conducted using the Protein ANNotation with Z-scoRE (PANNZER2) tool [92]. With the goal of analyzing cis-regulatory elements of the AP2/ERF genes in *S. scabra*, promoter sequences of these genes were first extracted using the GXF Sequence Extract tool in TBtools version 2.224 [90], with a parameter set to retrieve 2000 base pairs upstream of the *Ssc*AP2/ERF genes. Subsequently, these promoter sequences were submitted to the PlantCARE database (https://bioinformatics.psb.ugent.be/webtools/plantcare/html/ (accessed on 16 October 2024)), aiming to identify the presence of cis-regulatory elements in the promoter regions.

To identify potential transcription factors associated with the cis-regulatory elements present in *Ssc*AP2/ERF promoters, the promoter sequences were also analyzed using the MEME tool (https://meme-suite.org/meme/tools/meme (accessed on 25 October 2024)) to uncover up to 15 conserved motifs. The identified motifs were compared to the TOMTOM database [93]. The binding site profiles were retrieved from the JASPAR Core Plants database [94], which contains a comprehensive and curated collection of plant-specific transcription factors.

### 4.8. Prediction of Proteins’ Secondary and Tertiary Structures

For each subgroup of the AP2/ERF subfamily (I-X, AP2, and RAV), a representative was selected, prioritizing those encoded by genes expressed under the analyzed conditions. In cases where no differential expression was observed, the protein was chosen randomly. These proteins were used for predicting tertiary structures. Batch CD-Search tool (https://www.ncbi.nlm.nih.gov/Structure/bwrpsb/bwrpsb.cgi (accessed on 25 July 2025)) was used to remove signal peptides, aiming to preserve only the region corresponding to the domains. If the protein contained two domains, the region between the domains was retained. The resulting sequences were submitted to AlphaFold3 [95] with parameter set num_relax 5. The final model selected was rank_1, representing the best statistical model of the protein. The Visual Molecular Dynamics software—VMD version 1.9.3 [96], was used for visualization and editing of the modeled structures. Model validation was performed considering the QMEAN-DisCo Global Z-score values from the QMEAN-DisCo (https://swissmodel.expasy.org/qmean/ (accessed on 27 July 2025)), ProSA-web (https://prosa.services.came.sbg.ac.at/prosa.php (accessed on 27 July 2025)) programs, and Procheck (https://www.ebi.ac.uk/thornton-srv/software/PROCHECK/ (accessed on 27 July 2025)).

### 4.9. In Silico Expression Profile of SscAP2/ERF Transcripts and Quantitative Real-Time PCR

BLASTp of the *Ssc*AP2/ERF proteins genomically identified was initially performed against the translated *S. scabra* RNA-Seq transcriptomes, aiming to identify differentially expressed AP2/ERF transcripts. Only transcripts containing the AP2 domain were selected for differential expression analysis.

RNA-Seq gene expression was validated by real-time quantitative PCR (qPCR) following the MIQE guidelines (Minimum Information for Publication of Quantitative Real-Time PCR Experiments; [97]). Reactions were performed on a CFX96 Touch Real-Time PCR System (Bio-Rad, Hercules, CA, USA) with three biological and three technical replicates per sample. Detection was carried out using the intercalating dye SYBR Green. The thermal cycling protocol consisted of an initial denaturation step at 95 °C for 2 min, followed by 40 cycles of 95 °C for 15 s and 60 °C for 1 min. To verify the specificity of the amplified products, melting curves were generated from 65 °C to 95 °C at a heating rate of 0.5 °C s^−1^, with fluorescence acquisition every 0.3 °C. Amplification efficiency (E = 10(^−1^/slope)), correlation coefficient (R^2^), intercept (Y), and slope were determined using standard curves constructed from serial dilutions of an equimolar pool containing aliquots of all analyzed samples. The reference genes β-tubulin and ubiquitin were used to normalize gene expression data, as described by Ferreira-Neto et al. [23].

The primers were designed with Primer3Plus (https://www.bioinformatics.nl/cgi-bin/primer3plus/primer3plus.cgi (accessed on 15 July 2025)) with the following parameters: product size 70–150 bp, primer length 18–22 bp, primer melting temperature (Tm) 58–60 °C, max Tm difference 5.0 °C, CG clamp 1, and maximum self-complementarity and maximum 3’ self-complementarity of 4.0 (Supplementary Material, Table S3). Specificity primers were validated in silico using Primer-BLAST (https://www.ncbi.nlm.nih.gov/tools/primer-blast/index.cgi (accessed on 15 July 2025)). Relative expression levels of target transcripts were analyzed using the REST software in its standard mode [98]. Statistical significance of differences between experimental conditions was assessed by hypothesis testing, adopting *p* < 0.05 as the threshold for significance.

## 5. Conclusions

This study is the first to identify and characterize AP2/ERF transcription factors at genomic and transcriptomic levels in *Stylosanthes scabra*, assessing their behavior under water deficit conditions using an integrated multi-omics approach. The results provided relevant insights into the structural, functional, and transcriptional profiles of these genes, suggesting their central role in the plant’s adaptive response to water deficit stress. In silico expression analysis (RNA-Seq) revealed the upregulation of 21 candidate genes, which stand out as promising targets for applications in breeding programs aimed at targeting drought tolerance. Structural models of AP2/ERF proteins demonstrated high predictive accuracy, reinforcing the relationship between conformational variations and their biological functions.

The high number and high diversity of *Ssc*AP2/ERF (295) reflect the allopolyploid condition of this crop, followed by other duplication events subjected to purifying selection. Such an arsenal of *Ssc*AP2/ERF genes probably contributes to the adaptation of *S. scabra* to semiarid conditions of the Caatinga environment.

Our findings support previous studies on the importance of AP2/ERF factors in regulating responses to abiotic and biotic stresses, as well as their interaction with other molecular signaling pathways. The identification of cis-regulatory elements associated with adaptation to water stress, including DRE, MYB, TC-rich repeats, and MYC, strengthens the hypothesis that these genes play a key role in regulating gene expression under adverse conditions. Thus, the SscAP2/ERF genes identified in this study represent promising targets for biotechnological strategies—such as transgenics and gene editing—aimed at developing more resilient crops capable of withstanding climate change and water scarcity, thereby contributing to food security and agricultural sustainability. However, the functional roles inferred for these genes still require experimental validation. Approaches such as promoter::GUS assays, transient expression, gene silencing, or overexpression in model plants will be essential to confirm their regulatory functions and fully support their application in future breeding and biotechnological programs.

## Data Availability

The genomic and RNA-Seq raw reads substantiating the findings of this article are accessible in the NCBI Bioproject repository (https://www.ncbi.nlm.nih.gov/bioproject/ (accessed on 23 October 2025)), under the accession PRJNA924790, and in the NCBI SRA (https://www.ncbi.nlm.nih.gov/sra (accessed on 23 October 2025)) repository, under the accession PRJNA837909, respectively.

## Supplementary Materials

The following supporting information can be downloaded at: https://www.mdpi.com/article/10.3390/plants15010158/s1. Figure S1: Multiple sequence alignment of protein derived from genes in the DREB family containing positions 14 and 19, used to evaluate conserved and variable regions identified in this study; Figure S2: Multiple sequence alignment of protein derived from genes in the ERF family containing positions 14 and 19, used to evaluate conserved and variable regions identified in this study; Figure S3: Diversity of motifs identified in the DREB subfamily of AP2/ERF proteins from *S. scabra*. Motifs are represented by different colors according to the color code, and asterisks indicate motifs that are exclusive to the group; Figure S4: Diversity of motifs identified in the ERF subfamily of AP2/ERF proteins from *S. scabra*. Motifs are represented by different colors according to the color code, and asterisks indicate motifs that are exclusive to the group; Figure S5: Diversity of motifs identified in the AP2 and RAV subfamilies of *S. scabra*. Motifs are represented by different colors according to the color code, and asterisks indicate motifs that are exclusive to the group. Table S1: Summary of protein characteristics of the identified AP2/ERF genes; Table S2: Transcription factor binding motifs identified in promoter regions of *S. scabra* AP2/ERF genes, including their corresponding TF families, statistical significance, and sequence logos; Table S3: Primers used for validation of RNA-Seq data by real-time PCR of gene expression in *Stylosanthes scabra*. The sequences are shown in the forward and reverse orientations; Table S4: Validation in different software tools of the modeling of the 13 models used.

## Abbreviations

ABA (abscisic acid); ABRE (ABA-responsive element); AP2/ERF (APETALA2/Ethylene Responsive Factor); BLASTP (Basic Local Alignment Search Tool for Proteins); DREB (dehydration responsive element binding); ET (ethylene); GO (gene ontology); HMM (Hidden Markov Model); Ka/Ks (ratio of nonsynonymous to synonymous substitution rates); NCBI (National Center for Biotechnology Information); NJ (Neighbor Joining); TF (transcription factor); TFBS (transcription factor binding site).
