# Supplementary material for: Genome- and Transcriptome-Wide Characterization of AP2/ERF Transcription Factor Superfamily Reveals Their Relevance in Stylosanthes scabra Vogel Under Water Deficit Stress
Source: Plants (Basel). 2026 Jan 4;15(1):158. doi: 10.3390/plants15010158 (PMC12787715; doi:10.3390/plants15010158)
Supplement: Supplementary file 1 [file plants-15-00158-s001.zip › Supplementary_Figures_AP2.pdf]

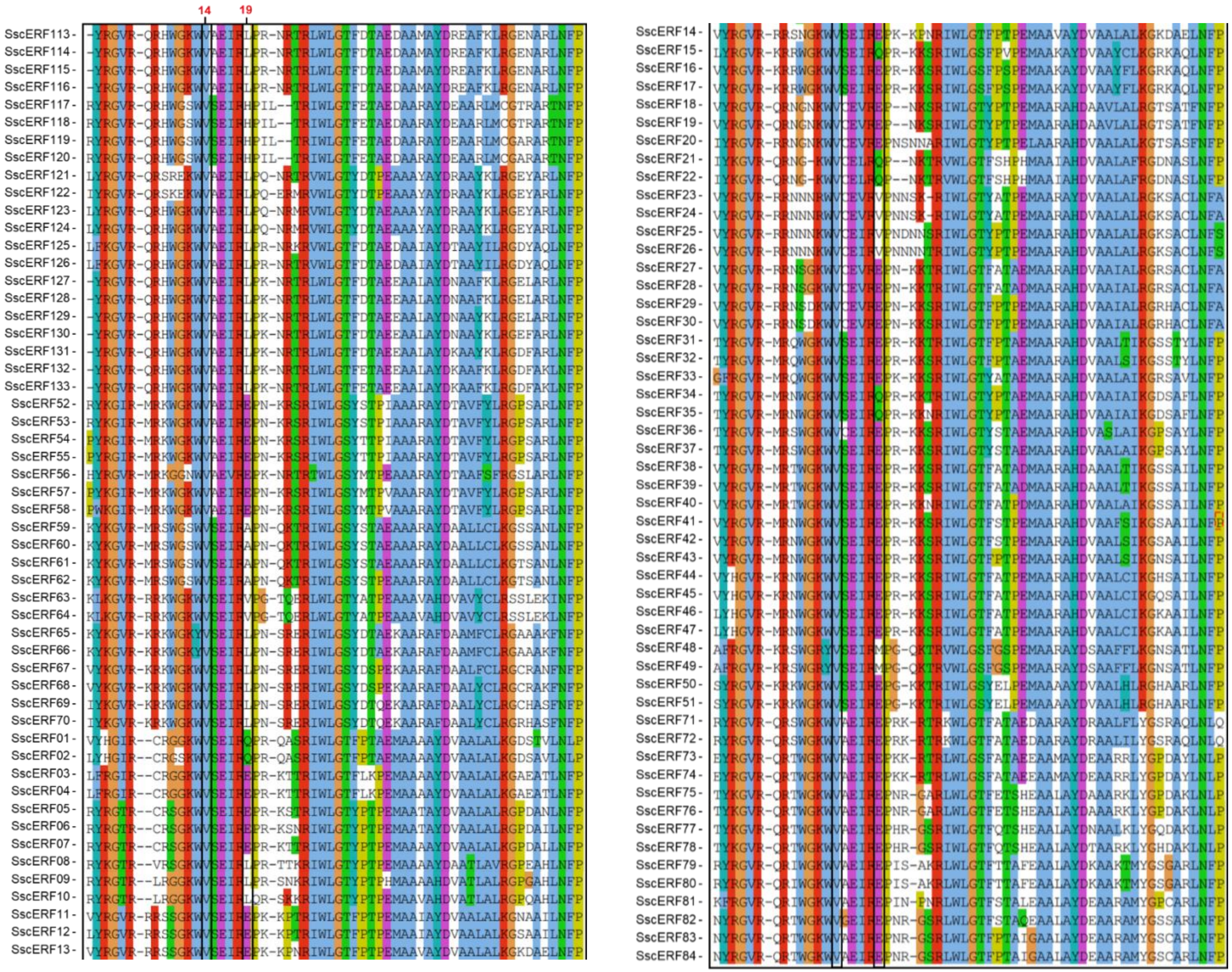

Figure S1. Multiple sequence alignment of protein derived from genes in the DREB family containing positions 14 and 19, used to evaluate conserved and variable regions identified in this study.

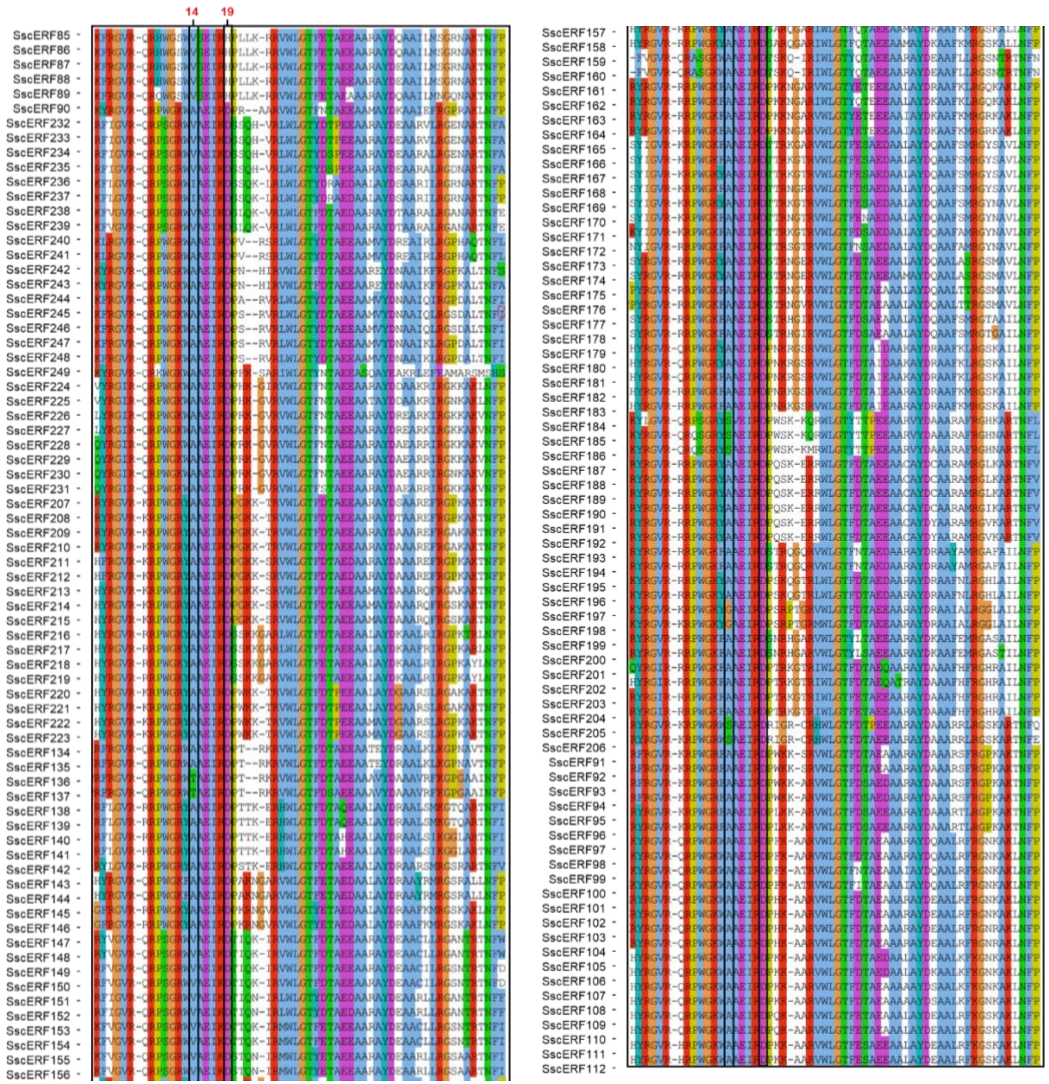

Figure S2. Multiple sequence alignment of protein derived from genes in the ERF family containing positions 14 and 19, used to evaluate conserved and variable regions identified in this study.

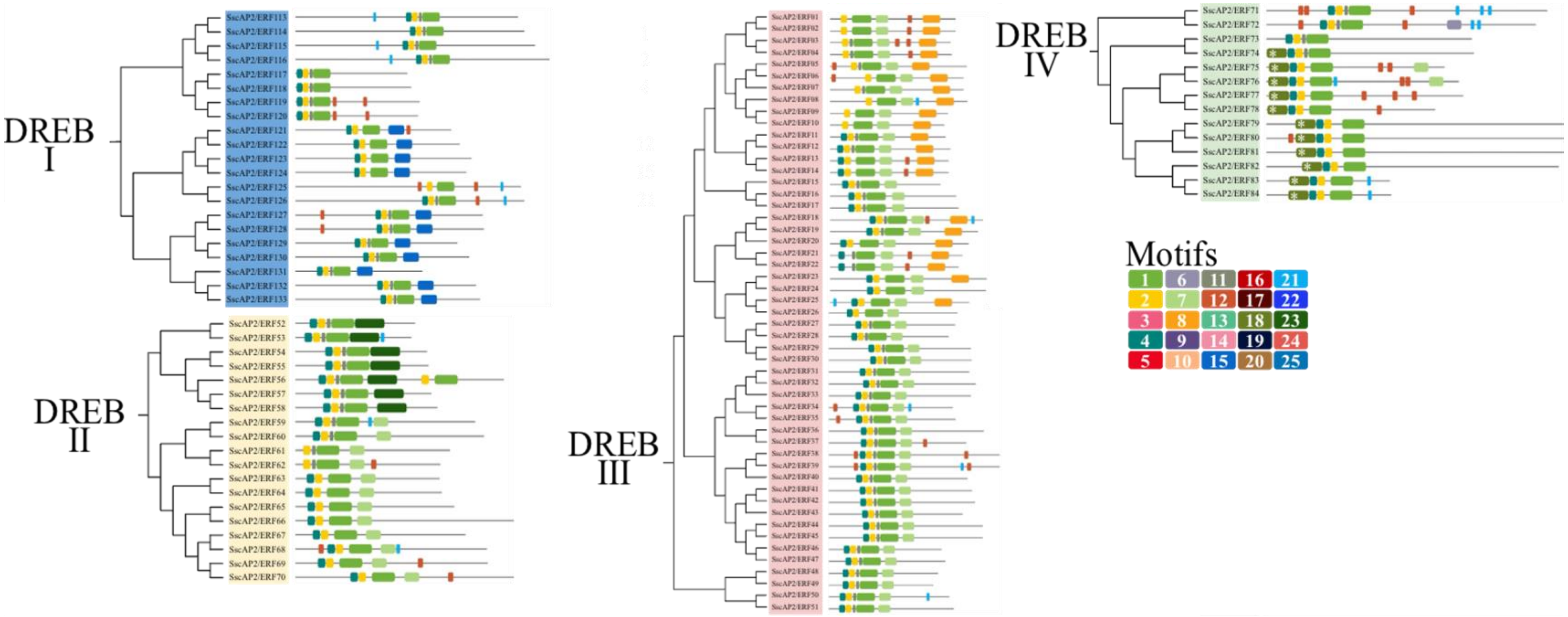

Figure S3. Diversity of motifs identified in the DREB subfamily of AP2/ERF proteins from *S. scabra*. Motifs are represented by different colors according to the color code, and asterisks indicate motifs that are exclusive to the group.

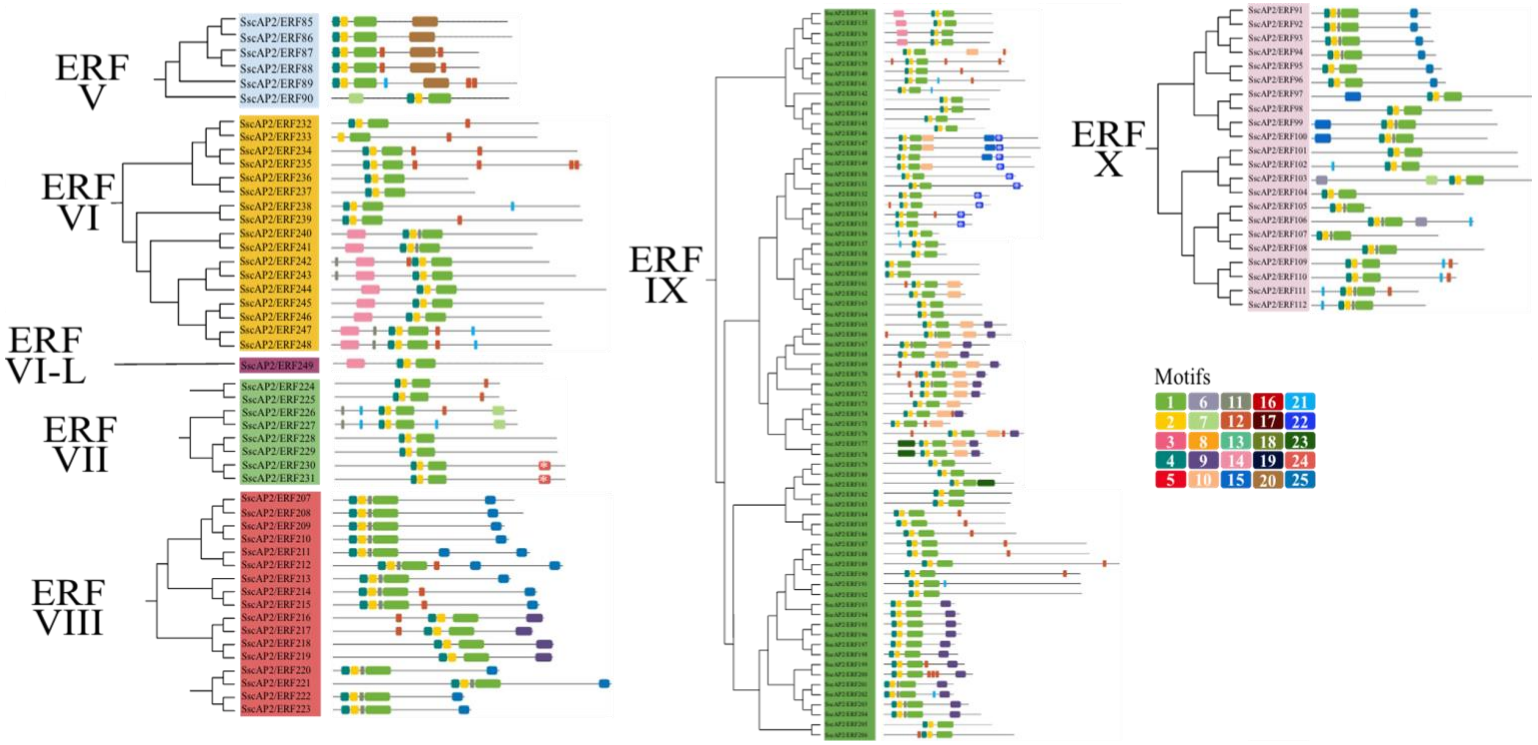

Figure S4. Diversity of motifs identified in the ERF subfamily of AP2/ERF proteins from *S. scabra*. Motifs are represented by different colors according to the color code, and asterisks indicate motifs that are exclusive to the group.

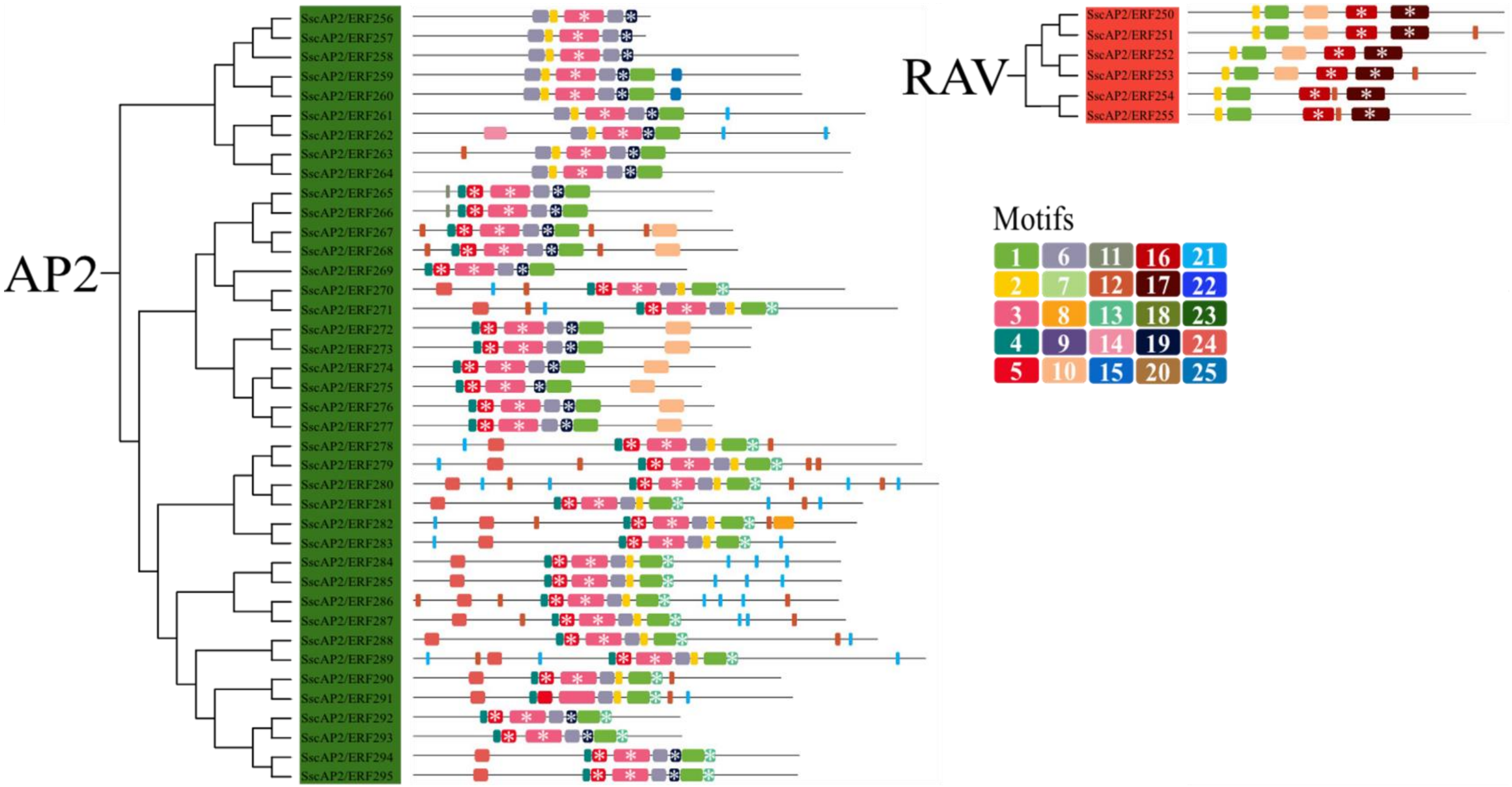

Figure S5. Diversity of motifs identified in the AP2 and RAV subfamilies of S. scabra. Motifs are represented by different colors according to the color code, and asterisks indicate motifs that are exclusive to the group.
